# Probiotic Lactobacilli in Fermented Dairy Products: Selective Detection, Enumeration and Identification Scheme

**DOI:** 10.3390/microorganisms9081600

**Published:** 2021-07-27

**Authors:** Nasim Farahmand, Labia I. I. Ouoba, Shahram Naghizadeh Raeisi, Jane Sutherland, Hamid B. Ghoddusi

**Affiliations:** Microbiology Research Unit, School of Human Sciences, London Metropolitan University, London N7 8DB, UK; n.farahmand@londonmet.ac.uk (N.F.); ouobairene@hotmail.com (L.I.I.O.); shahram9112006@yahoo.com (S.N.R.); jane@sutherland.mobi (J.S.)

**Keywords:** probiotic, lactobacilli, fermented dairy product, identification, enumeration, rep-PCR

## Abstract

A selection of 36 commercial probiotic fermented dairy products from UK and Europe markets were evaluated for the numbers, types, and viability of *Lactobacillus* strains against the stated information on their packages. A comparative study was carried out on selectivity of MRS-Clindamycin, MRS-Sorbitol, and MRS-IM Maltose, to select the right medium for enumeration of probiotic *Lactobacillus*. Based on selectivity of medium for recovery of the targeted lactobacilli, and also simplicity of preparation, MRS-Clindamycin was chosen as the best medium for enumeration of probiotic *Lactobacillus* in fermented milks. The results of enumeration of lactobacilli showed that 22 out of a total 36 tested products contained more than 10^6^ colony-forming units/g at the end of their shelf life, which comply with the recommended minimum therapeutic level for probiotics. Rep-PCR using primer GTG-5 was applied for initial discrimination of isolated strains, and isolates, which presented different band profile, were placed in different groups. The isolated *Lactobacillus* spp. were identified mainly as *Lactobacillus acidophilus*, *Lactobacillus casei,* and *Lactobacillus paracasei* by analysis of partial sequences of the 16S ribosomal RNA and *rpoA* genes.

## 1. Introduction

Certain dairy products are vehicles by which consumers receive adequate counts of probiotic lactobacilli [1]. Probiotic effects are dependent on the number of viable microbial cells that reach the human gut [2]. Therefore, their viability in the product is considered as an important prerequisite for achieving health effects. 

There are various reports regarding the adequate number of probiotic microorganisms in different products in order to ensure the probiotic effects. The recommended quantity of probiotic lactobacilli that needs to be consumed for a health benefit varies in different studies [3]. Some of the suggested minimum levels of viable cells in dairy products are 10^5^ CFU/g [4], 10^6^ CFU/g [5,6], and 10^7^ CFU/g [7]. It is not simple to keep a high number of viable probiotic bacteria in fermented milk throughout the shelf life, because their viability in the product matrix is influenced by numerous factors. Such parameters include temperature of storage condition, hydrogen peroxide (H_2_O_2_), which might be produced by other existing bacteria, dissolved oxygen content due to process conditions, pH of the final product and, finally, strain variation, which may be considered the most important factor for the survival of probiotic cultures in the final product [8].

Probiotic lactobacilli are incorporated alone or in combination with other commercial cultures into specific dairy products. Interactions between microorganisms in cocultured products cause difficulties in enumeration. *Lactobacillus acidophilus, Lactobacillus casei,* and *Bifidobacterium lactis* are the most frequently used strains in commercial probiotic products [9].

In the past few decades, many selective/differential media have been developed for accurate enumeration of *Lactobacillus* spp. in fermented milks. However, due to presence of closely related species of *Lactobacillus* spp. in probiotic products, the differential enumeration seems challenging and relies directly on differences in colonial morphology [10].

There are also various instructions regarding the probiotic enumeration, but only a few are official protocols for lactobacilli, for example, ISO (2006). Enumeration in cocultured products is more complicated than in products made with single culture. In mixed cultures, inhibitory agents are needed to suppress the interfering species in order to recover the target lactobacilli. However, one real concern is that some culture media that contain antibiotics might also restrict the growth of target lactobacilli, and the counts may not be representative of the real number of viable cells present in the product [11]. On the other hand, some antibiotics cannot inhibit the growth of all nontarget bacteria [12]. Several reports have revealed the misidentification of a number of strains belonging to some lactobacilli [13,14].

The probiotic ability is often strain dependent and, therefore, accurate detection and identification of probiotic lactobacilli is required. Characteristics including phenotype, physiological and biochemical features, and sequence comparisons of 16S rRNA gene have been suggested to make the identification of *Lactobacillus* species more reliable [15]. There are, however, taxonomic dispute and ambiguity among some lactobacilli due to the differences at nucleotide level in the 16S rRNA gene [16]. It is therefore hard to differentiate between some species and strains of lactobacilli [17], and some closely related groups of lactobacilli species are indistinguishable based on phenotype. Molecular identification methods, on the other hand, have proven to be consistent, rapid, reliable, and reproducible, compared to phenotypic methods. For example, species-specific oligonucleotide probes have been employed to identify various *Lactobacillus* species [18]. Most genetic probes have been designed based on 16S rRNA or 23S rRNA genes [19]. 

In general, there are some ambiguities in differentiation of specific lactobacilli. According to the study by Singh et al. (2009), there are similarities at nucleotide level in the 16S rRNA gene in some lactobacilli, such as *Lb. acidophilus*, *Lb. casei*, *Lb. plantarum,* and *Lb. delbrueckii*, making it hard to distinguish them in a mixed culture. It has been reported that sometimes *Lb. gasseri* and *Lb. johnsonii* are difficult to differentiate from each other, even by molecular methods [20]. *Lactobacillus plantarum* and *Lb. pentosus* have greater than 99% similarity with only 0.3% difference in their 16S rRNA sequences [21]; however, some alternative molecular markers have been used for discrimination among these species. 

Recent research into the relatedness of species in the *Lb. acidophilus* group has used sequence analyses of genes such as 16S rRNA, *rpoA*, *pheS* [22], *groEL* [23], and *tuf* [24].

The aim of the work described in this research was to isolate, enumerate, and identify *Lactobacillus* spp. in commercial probiotic dairy products from the UK and European supermarkets using genotyping methods. In addition, accuracy of the label descriptions for fermented milk products was assessed.

The study was carried out before the introduction of the new taxonomy for the *Lactobacillaceae* family in April 2020, and all the original old bacterial names kept unchanged.

## 2. Materials and Methods

### 2.1. General/Selective/Elective Media

MRS agar (CM0361, Oxoid, Basingstoke, UK) was used as general medium. MRS agar supplemented with 0.1 mg L^−1^ clindamycin (C5269, Sigma, Poole, UK) was prepared according to ISO (2006) for enumeration of *Lb. acidophilus, Lb. rhamnosus, Lb. casei,* and *Lb. paracasei*. MRS agar was supplemented with 20 g L^−1^ sorbitol [25] to replace the original dextrose for elective enumeration for *Lb*. *acidophilus*. MRS-IM Maltose agar [26] was used for elective differential enumeration of *Lb*. *acidophilus* and *Lb. casei.* All elective or selective supplements were purchased from Sigma (Poole, UK).

### 2.2. Microbial Culture

Three commercial cultures (*Lb. acidophilus* La5, *Lb. delbrueckii* subsp. *bulgaricus* Lb12, and *Lb. casei* C431) were kindly provided by Chr. Hansen. Type strain *Lb. delbrueckii* subsp. *bulgaricus* 11778, *Lb. acidophilus* 701748, *Lb. casei* subsp. *casei* 11970, and *Lb. paracasei* subsp. *paracasei* 700151 were purchased from National Collections of Industrial, Marine and Food Bacteria (NCIMB).

### 2.3. Commercial Probiotic Products

Thirty-six commercial fermented milks claiming to contain probiotic *Lactobacillus* strains were purchased from UK and European supermarkets, transported to the laboratory, and stored at 4 °C. Samples from countries outside the UK were purchased and sent to the UK in a cool box. Table 1 shows details of the tested products.

### 2.4. Measurement of pH Value 

The pH of the initial and final (on the expiry date) samples of the fermented milks was measured using a Whatman PHA 2000 pH meter.

### 2.5. Determination of Viable Cell Count of Lactobacillus Spp. in the Fermented Milks

Four pots of each product were purchased. All products were analysed on the day of purchase (two pots) and again on their expiry date (two pots) using unopened product each time. One gram of homogenised sample was mixed with 9 mL of Maximum Recovery Diluent (MRD) (CM0733, Oxoid, Basingstoke, UK) and vortexed. Dilutions up to 10^−8^ were made using MRD. Agar plates were divided into four sections using a marker, and 25 µL of each dilution was spread onto each quarter of MRS, MRS-IM Maltose, MRS-Sorbitol, and MRS-Clindamycin in duplicate. The plates were then incubated for three days at 37 °C in an anaerobic cabinet (Don Whitley, Skipton, UK) using an atmosphere of 80% nitrogen, 10% hydrogen, and 10% carbon dioxide.

### 2.6. Isolation and Storage of the Isolates

Two to four typical colonies grown on MRS-Clindamycin were randomly harvested from each product and streaked on MRS agar. Following overnight anaerobic incubation at 37 °C, the single colonies were streaked on MRS agar for the second time and incubated in the same conditions. One pure isolated colony was picked up and inserted aseptically into a cryovial (Micro bank, Pro-Lab Diagnostics, Neston, UK), following manufacturer’s instructions, and stored at −20 °C. 

### 2.7. Grouping and Identification of Isolates 

#### 2.7.1. DNA Extraction

Fresh colonies of isolates were grown from cryovial beads following two consecutive streaks on MRS agar. The DNA was extracted using InstaGene (Bio-Rad, Hemel Hempstead, UK), according to the manufacturer’s instructions, and stored at −20 °C.

#### 2.7.2. Differentiation (Grouping) of the Isolates Using Rep-PCR

Repetitive element sequence-based polymerase chain reaction (Rep-PCR) was applied for differentiation of isolates by the method of Ouoba et al. (2008) [27]. Rep-PCR was undertaken in 25 µL of reaction mixture containing 2 µL of DNA template, 2.5 µL of 10 × PCR buffer (Applied Biosystems, UK), 4 µL of dNTP (1.25 mmol L^–1^; Promega, UK), 2 µL of MgCl_2_ (25 mmol L^–1^; Applied Biosystems, UK), 4 µL of GTG-5 (5 pmol µL^–1^) primer (GTG-GTG-GTG-GTG-GTG), 2.5 U of Taq polymerase (5 U µL^−1^; Applied Biosystems, UK), and 10.25 µL of autoclaved high-purity water (Sigma, Poole, UK). Amplification consisted of 30 PCR cycles in a thermocycler (GeneAmp PCR 2700 system). The cycling was programmed as follows: initial denaturation at 94 °C for 4 min followed by 30 cycles of denaturation at 94 °C for 30 s, annealing at 45 °C for 1 min and elongation at 65 °C for 8 min. In addition, final extension at 65 °C for 16 min ended the rep-PCR, and the amplified product was cooled at 4 °C. The DNA fragments were separated by applying 10 µL of each PCR product with 2 µL of loading buffer (Sigma, Poole, UK) on a 1.5% agarose gel (BioRad, Warford, UK). A DNA molecular marker (Sigma, Poole, UK) was included as standard for the calculation of the size of the fragments. The gel was run in 1 × Tris–Borate–EDTA (TBE) buffer (Sigma, Poole, UK) for 2 h at 120 V, and photographed using a UV transilluminator. The DNA profiles were observed, and all bacteria showing the same profile were clustered in the same group by combined visual observation, as well as cluster analysis using the Bio-Numerics system: BIO-NUMERICS 2.50: Dice’s Coefficient of similarity with the unweighted pair group method with arithmetic averages clustering algorithm (UPGMA; Applied Maths, Saint-Martens-Latem, Belgium). 

#### 2.7.3. Identification of the Isolates by Sequence Analysis of 16S Ribosomal RNA Gene

Following rep-PCR screening and arranging the isolates into different groups, further identification was carried out using 16S rRNA gene sequencing, according to the method described by Ouoba et al. (2008). 

A search was performed in the GenBank database using the Blast program (National Center for Biotechnology Information, Bethesda, MD, USA). Sequences of representative isolates from each rep-PCR group were compared with the GenBank/DDBJ Nucleotide Sequence Data Libraries.

#### 2.7.4. Identification of Bacteria by rpoA Gene Sequencing

Primarily, all randomly selected isolates were identified by 16S rRNA gene sequencing; however, where it was not possible to distinguish between closely related species (i.e., *Lb. casei* and *Lb*. *paracasei*), amplification and sequencing of *rpoA* gene was carried out. 

The amplification of *rpoA* gene was carried out using the forward primer *rpoA*-21-F (5′ATG ATTC GAGA TTT GAA AAA CC 3′) and reverse primer *rpoA*-23-R (5′ACACT GTGA TTGA ATD CCGAT GCGA CG 3′) [28].

### 2.8. Statistical Analysis

All data were analysed statistically using SPSS version 20.0 (SPSS Inc., 444 North Michigan Ave., Chicago, IL, USA.). The two-tailed unpaired Student’s *t*-test was performed to determine differences at levels of significance of *p* < 0.05. Experiments were replicated at least three times.

## 3. Results

### 3.1. Enumeration of Lactobacillus Spp. in Commercial Fermented Milk

**Figure 1:**
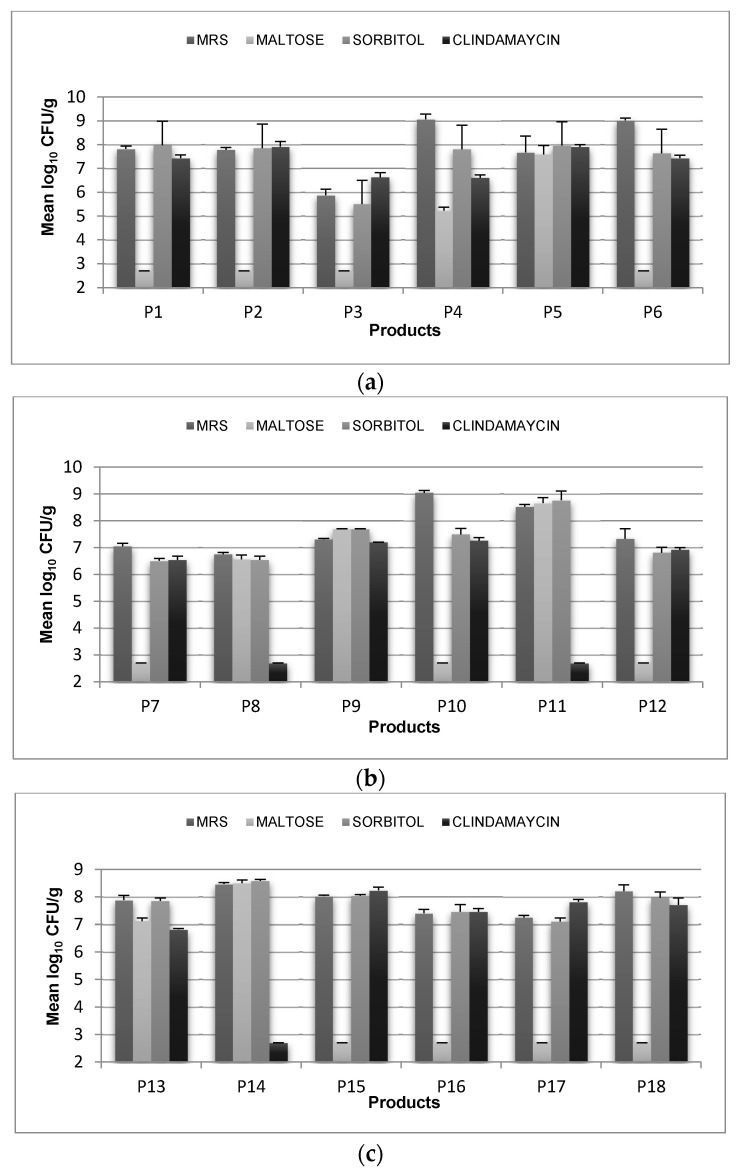

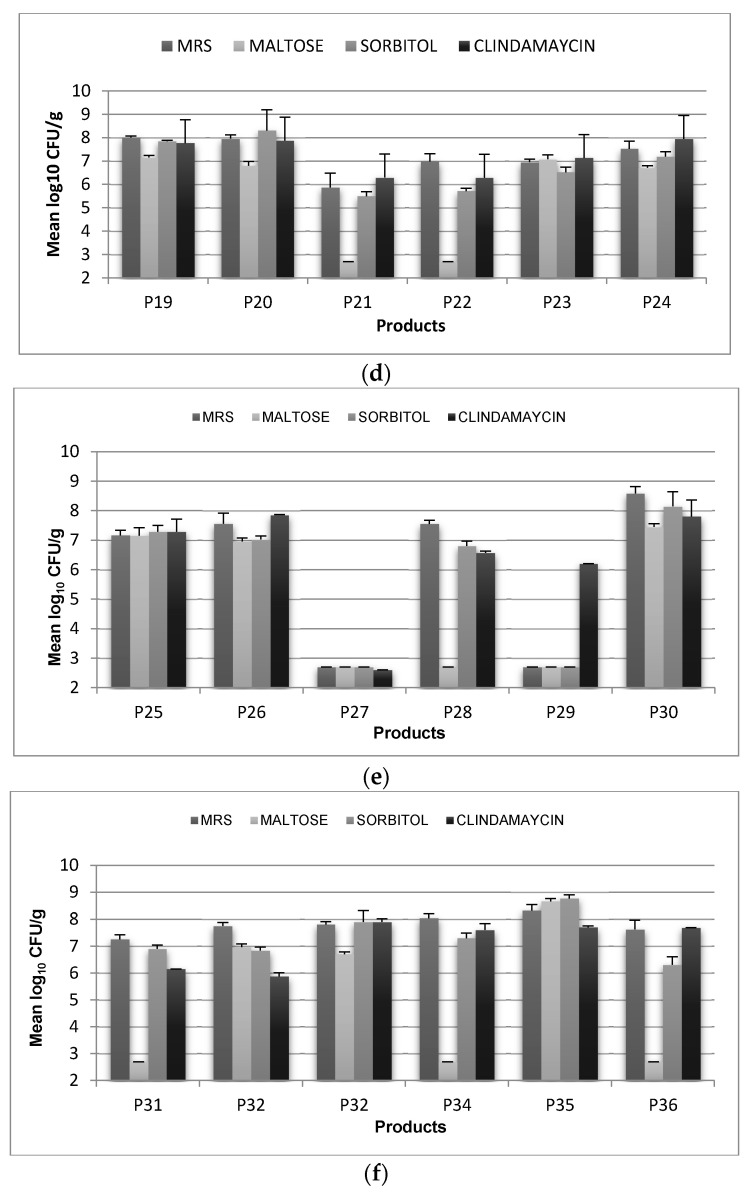
Bacterial count of presumptive *lactobacillus* spp. strains (log_10_ CFU/g) on (MRS, MRS Maltose, MRS-Sorbitol, MRS-Clindamaycin) at 37 °C after 48 h anaerobic incubation. Data are means ± SD (n = 4). P1–P36 are sample codes for tested probiotic products.

In the present study, MRS agar, MRS-IM Maltose agar, MRS-Sorbitol agar, and MRS-Clindamycin agar were used for enumeration of probiotic lactobacilli in 36 probiotic dairy products (Figure 1a–f). MRS agar was used as a nonselective reference medium. MRS-IM Maltose, MRS-Sorbitol, and MRS-Clindamycin are quite common as selective and elective media for counting of *Lactobacillus* species. The shape and size of colonies of *Lactobacillus* species vary on different media. An interesting observation was that on MRS-Clindamycin, *Lactobacillus acidophilus* gives star shaped, irregular small colonies, and *Lb. casei* gives larger, regular colonies on MRS-Clindamycin. *Lactobacillus casei* colonies on MRS sorbitol, MRS-IM Maltose agar, and even MRS agar had regular shape with no difference to *Lb. acidophilus.* This makes the MRS-Clindamycin also serve as a differential agar. *Lactobacillus acidophilus* forms small, rough, brownish, dull colonies of 0.1 to 0.5 mm on MRS-Sorbitol agar, which was very difficult to differentiate from *Lb. casei.*

Generally, MRS-IM Maltose agar did not give a good recovery of the lactobacilli, even when compared with the control medium (MRS agar) and the other MRS variants. In this medium, 19 samples had lower than the estimated detection limit (log_10_ 2.7 CFU/g). Therefore, it was not considered as a suitable medium due to low recovery of the lactobacilli. 

MRS-Sorbitol showed higher viable counts than MRS-Clindamycin. Recovery of lactobacilli below the noted detection limit (log_10_ 2.7 CFU/g) was seen on MRS-Sorbitol and MRS-Clindamycin in two and three samples, respectively.

Comparison of the results indicated that in eight products (P8, P9, P11, P13, P14, P31, P32, and P35), the viable counts on MRS-Sorbitol were higher than on MRS-Clindamycin, while in six products (P15, P17, P26, P29, P34, and P36), viable counts on MRS-Clindamycin were higher than on MRS-Sorbitol. 

Thirty-one out of 36 fermented milks contained more than log_10_ 6 CFU/g on at least one medium at the time of purchase (Figure 1a–f).

**Figure 2:**
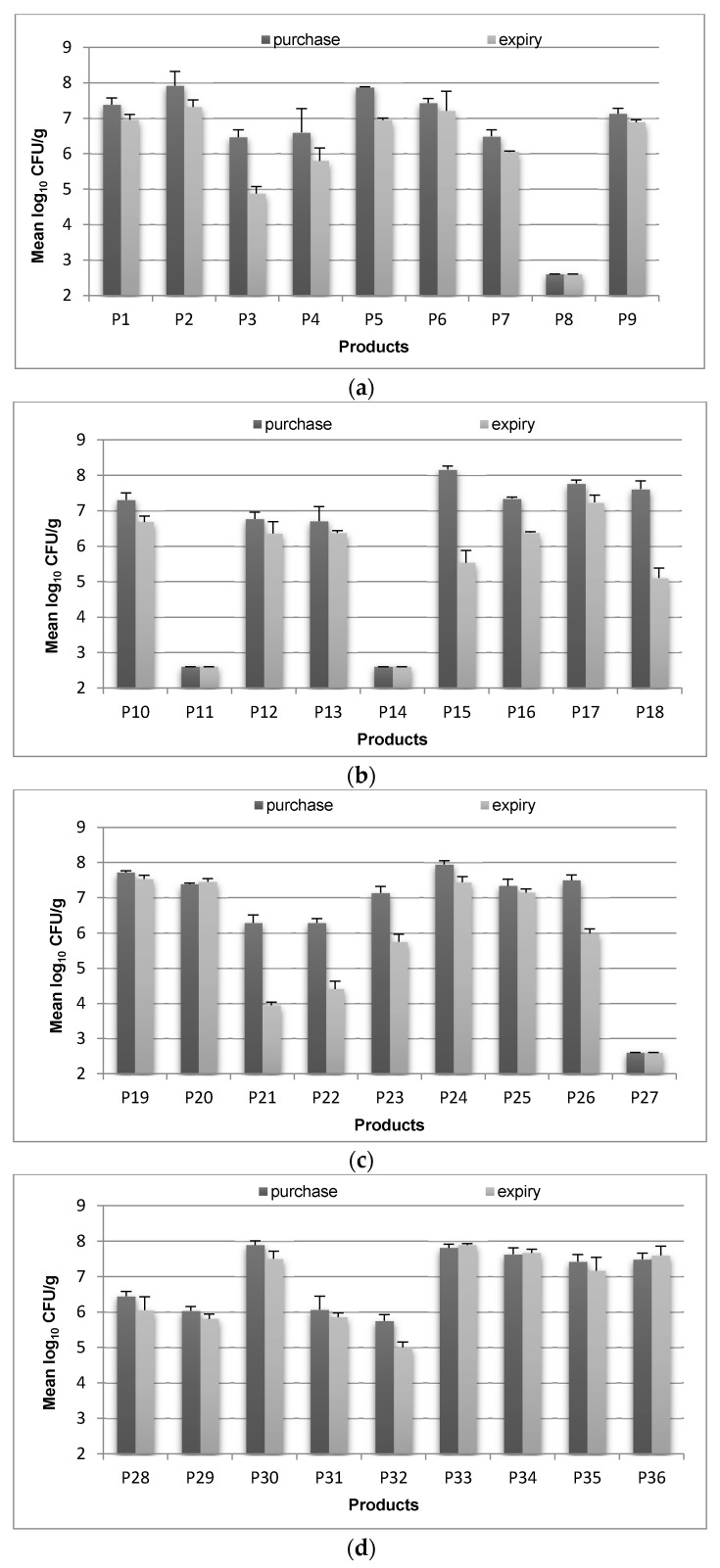
Bacterial count of presumptive *Lactobacillus* spp. strains (log_10_ CFU/g) on MRS-Clindamycin agar in tested products at the time of purchase and at the end of expiry date at 37 °C after 48 h anaerobic incubation. Data are means ± SD (n = 4). P1–P36 are sample codes for tested probiotic products.

The number of *Lactobacillus* recovered on MRS-Clindamycin agar at the expiry dates compared to the purchase dates are shown in Figure 2a–d. The number of *Lactobacillus* spp. declined almost in all samples. The highest decline was log_10_ 2.62 CFU/g in products P15 and P18. However, at the end of the shelf life, 22 (61.1%) of the tested samples contained greater than log_10_ 6 CFU/g of the product.

Out of the remaining 14 with less than log_10_ 6 CFU/g, products P3, P4, P15, P18, and P21–23 contained an initial *Lactobacillus* spp. population of more than log_10_ 6 CFU/g, which had significantly decreased to less than log_10_ 6 CFU/g by the expiry date (*p* < 0.05). However, products P8, P11, P14, P27, and P32 contained less than log_10_ 6 CFU/g viable *Lactobacillus* spp. at the time of purchase. Based on these results, even though MRS-Clindamycin did not perform better than MRS-Sorbitol, it was selected for further studies mainly because it was recommended by ISO (2006) and because the differentiation of *Lb. acidophilus* and *Lb. casei* was possible on this medium (morphology of the colonies were distinctively different).

### 3.2. Differentiation of Isolates by Rep-PCR

**Figure 3:**
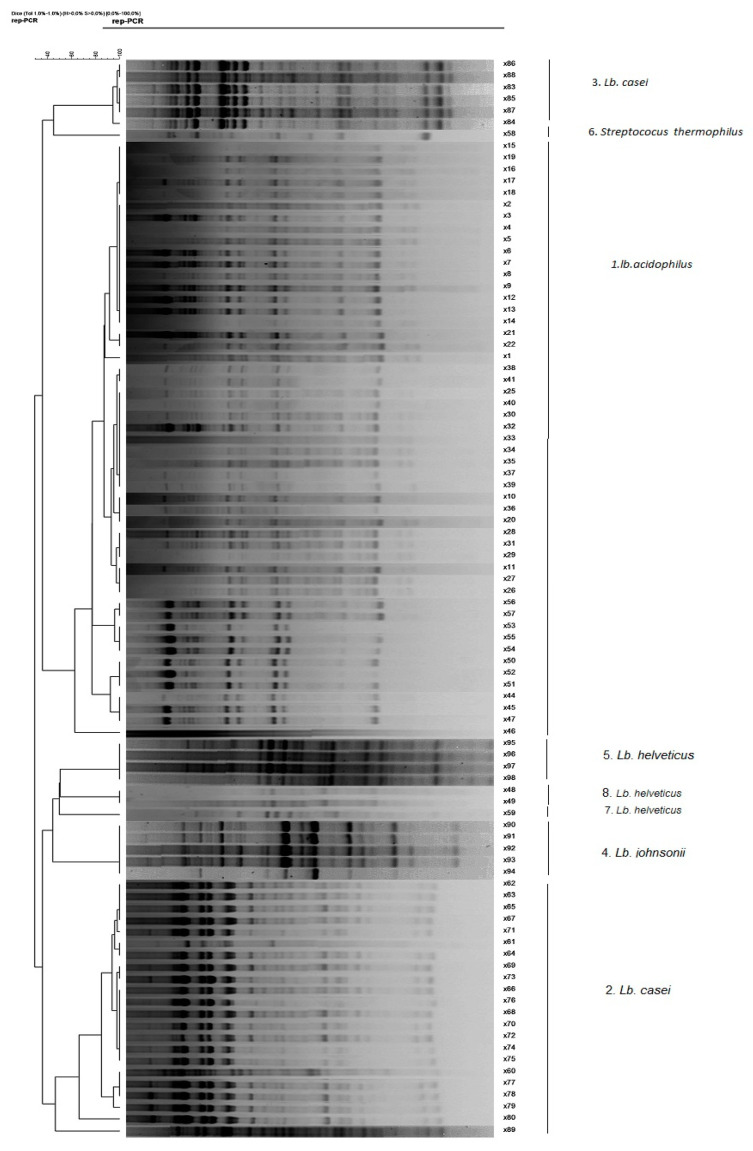
Dendrogram generated after cluster analysis of rep-PCR fingerprints of tested isolates. The ruler on the top left corner of the image indicates the similarity percentage.

A total of 85 isolates were selected from different media based on their shape, size, and/or colour. These isolates, along with the commercial and type strain *Lactobacillus,* were grouped using rep-PCR resulting in eight groups (Figure 3). Group A, as the major group, contained 51 isolates with the same DNA profile. Other groups included group B (22), C (6), D (5), E (4), F (1), G (1), and H (2) isolates.

In total, 20 isolates representative of groups A–H (corresponding to species 1–8 in Figure 3) were randomly selected from the above groups, and identified by partial sequencing of 16S rRNA and *rpoA* genes.

### 3.3. Identification of Isolates by Partial Sequencing of 16S rRNA and rpoA Genes

Random representatives of each group; A (6), B (3), C (4), D (2), E (2), F (1), G (1), and H (1) were analysed using the 16S rRNA gene, and further experiments with *rpoA* gene sequencing were applied when 16S rRNA gene failed to provide accurate identification. Table 2 presents the results of identification using 16S rRNA and *rpoA* gene sequencing of the tested isolates, compared with the identities claimed on the product labels.

The isolates from group A were all identified as *Lb. acidophilus,* and isolates from group B were identified as *Lb. casei/paracasei*. As the 16S rRNA gene sequencing could not differentiate between *Lb. casei* and *Lb. paracasei*, sequencing of *rpoA* gene was used to discriminate between these two species. However, *rpoA* gene sequencing also could not differentiate between these two closely related species. 

Similarly, isolates from group C were identified as *Lb. casei/paracasei* by both 16S rRNA and *rpoA* gene sequencing.

Isolates from group D were identified as *Lb. johnsonii* and group E as *Lb*. *helveticus*/*gallinarum*/*suntoryeus,* and *rpoA* gene sequencing could not differentiate between them. The only isolate from group F was identified as *Streptococcus thermophilus*. Groups G and H were identified as *Lb. helveticus/gallinarum/suntoryeus* by both 16S rRNA and *rpoA* gene sequencing.

Sequencing of *rpoA* gene in addition to sequencing of 16S rRNA was not able to discriminate between isolates from groups B, C, E, G, and H. Therefore, the DNA profiles of unconfirmed isolates were compared with those of type strains, and their identity confirmed according to their similarities with the type strains (Figure 3).

### 3.4. PH Reduction during the Shelf Life

The pH of most samples slightly declined during the cold storage until the end of their shelf life (Table 2). In one sample (product no. 20), however, pH value dropped by 0.42. While post-acidification by lactobacilli under cold storage is normal, it is not known why only in this sample the reduction was higher than the rest of the samples. 

## 4. Discussion

The use of food as a carrier for probiotic organisms is of considerable interest to food manufacturers due to the claimed health-associated benefits of probiotics. However, maintaining high numbers of viable probiotics in fermented milks is not easy, and a large quantity of probiotic cultures is needed to compensate for the likely losses of probiotics during the shelf life [29]. Procedures for enumeration of lactobacilli have not been properly defined. Such a situation causes difficulties in quality control of the probiotic products containing *lactobacillus* species using the conventional enumeration technique. The suitability of various media to selectively enumerate lactobacilli has been examined in different studies. Although there are several elective/selective media for isolation of lactobacilli, the levels of recovery of the lactobacilli are discordant with each other.

Oberg et al. (2011) reported that while MRS-Sorbitol is a medium designed for *Lb. acidophilus* in which sorbitol is the sole sugar, *Lb. casei* can also grow on the medium, although only at elevated incubation temperature (42 °C). At this temperature, the MRS-Sorbitol medium gave higher bacterial counts compared to the *Lb. casei* specific medium (*Lactobacillus casei* agar), indicating that it could be used to obtain the total LAB count at different temperature [30]. However, in our study, colonies of target strains were recovered at 37 °C on MRS-Sorbitol agar. Due to the high recovery, no other recovery temperatures were employed. 

MRS-Sorbitol demonstrated higher viable counts than MRS-Clindamycin, suggesting that MRS-Sorbitol might allow the growth of additional LAB. Shah (2000) stated that MRS-Sorbitol agar could not be used for selective enumeration of *Lb. casei* and *Lb. acidophilus* in products containing both bacteria.

This study also reports that MRS-IM Maltose is not an ideal choice for selective enumeration of lactobacilli since the recovery was low compared with other MRS variants.

MRS-Clindamycin has been proposed for enumeration of lactobacilli in different studies [10,11]. Furthermore, the International Organization for Standardization (ISO) (2006) recommended MRS-Clindamycin agar for the enumeration of *Lb. acidophilus* in dairy products in the presence of other probiotics including other lactobacilli, streptococci, and bifidobacteria [11]. Simplicity of medium preparation and availability of the antibiotic supplement led to its consideration as the preferred medium compared to the other selective media. Moreover, for *Lb. casei* to grow on MRS-Sorbitol, the incubation temperature should be raised to 42 °C, therefore it is impossible to have differentiation on one medium and at one incubation temperature [30]. Hence, in our research, MRS-Clindamycin was considered as a reliable medium to selectively enumerate *Lactobacillus spp.* in fermented dairy products. Having said that, the selectivity of MRS-Clindamycin may not be 100%, as *S. thermophilus,* which is difficult to distinguish morphologically from *Lactobacillus* spp., was also isolated and identified in sample no. 23. This was not further investigated.

Our research shows that on the purchase and the expiry dates, respectively, 86% and 61% of tested samples contained the minimum recommended therapeutic level of log_10_ 6–7 CFU/g, concordant with the findings of the others [29]. Other researchers have also reported commercially probiotic dairy products with inadequate amounts of viable probiotics [31,32,33], which in some cases may be attributable to disruption of the cold chain [34]. In this study, during cold storage, the number of *Lactobacillus* spp. in some samples decreased considerably. The most important contributing factors for loss of cell viability are decreasing pH during storage, presence of dissolved oxygen, and presence of preservatives in the final products [8]. In this study, the pH decline between the purchase and expiry date was in some cases noticeable. It could be due to continued fermentation process by LAB even in low temperatures (post-acidification). However, no correlation was found between pH decline of samples and their probiotic counts.

The presence of dissolved oxygen might be the other important reason for drop in viability of cell count in fermented milk [35]. The majority of tested products in this study were stirred yoghurts, in which air could have been incorporated when the yoghurt was mixed with the fruit compote. In addition, some of the commercial fruit products contain preservatives to control contamination and this might affect the viability of the probiotic cells [36].

Based on results obtained in this research, which confirmed lower counts of probiotic cultures approaching the end of shelf life, and supported by the study of Jayamanne and Adams (2006), it is recommended that probiotic fermented products need to be consumed earlier than the expiry date to ingest maximal numbers of probiotic bacteria. 

Although there are no universally established standards for microbial content and health claims for probiotic products, the manufacturers should at least clearly express the genus, species, and strain of the probiotic microorganism(s) and also the minimum viable count of each probiotic strain at the end of shelf life [3,37]. To ensure that the consumers benefit from commercial probiotic products, it is necessary to confirm the identity of the claimed organisms at species/strain level and that they are present in the product in appropriate numbers before consumption. Some of the tested products in this study presented inadequate information on the labels. Microbial investigations of probiotic products by others have indicated that the number and identity of recovered species do not always correspond to those stated on the labels of products [38,39].

Identification of probiotic species used in carrier products should be verified in support of claimed health benefits. To obtain accurate and reliable identification of the probiotic species, molecular techniques should be applied. It has been suggested that DNA profiling by PCR-based methods are the best means for identification of probiotic bacteria at strain level [9,40]. Many misidentifications of probiotic microorganisms may be due to the use of solely phenotypic methods for taxonomic characterization [41].

The rep-PCR fingerprinting profile revealed relative genetic differences between the tested isolates. In this study, 85 isolates from fermented milks were grouped based on their DNA patterns by rep-PCR, and 20 isolates out of 85 were selected for identification by sequence analysis of 16S rRNA. Amplification of the 16S rRNA gene often provides a rapid and reliable tool for bacterial identification without the need for phenotypic characterization. However, 16S rRNA sequencing cannot discriminate between closely related species. Thus, sequencing of alternative genes, such as *rpoA,* with more discriminatory power has been proposed [42,43].

In this research, amplification and sequencing of the *rpoA* gene did not provide enhanced discriminatory information for the tested isolates compared to the use of 16S rRNA gene sequences. Sequencing of other genes, such as *rpoB* and *pheS,* would enhance discriminatory potential, enabling differentiation of strains with close genetic profiles. Anyogu et al. (2014) stated that sequencing of the *pheS*, *rpoA,* and *rpoB* genes along with 16S rRNA gene sequencing provides a better identification of LAB and *Bacillus* isolate.

Even though more media have been suggested in recent years for the enumeration of probiotic lactobacilli in fermented dairy products, none seems to be suitable for all lactobacilli or at least for *Lb. acidophilus*/*Lb. casei* (which are the two most frequently used lactobacilli in the products marketed in the UK/EU), or at the same time be able to act as a differential medium for these two species. Therefore, in this study we examined and compared a limited number of media. 

## 5. Conclusions

Evaluation of MRS-IM Maltose, MRS-Sorbitol, and MRS-Clindamycin as selective media for enumeration of probiotic *Lactobacillus* spp. in commercial fermented milks indicated that MRS-IM Maltose and MRS-Sorbitol were not the best choices for enumerating lactobacilli in fermented dairy products. Instead, the advantage of MRS-Clindamycin was its simplicity and ease of preparation, as well as being differential for *Lb. acidophilus* and *Lb. casei*. Our study of commercial probiotic dairy products in the UK/European market has shown that the most frequent species used in the probiotic products was *Lb. acidophilus* followed by *Lb. casei*. Some other strains were identified which are not popular in fermented dairy products. Commercial use of other useful probiotics, such as *Lb. helveticus*, *Lb. plantarum,* and *Lb. fermentum,* is recommended for dairy producers to provide more diversity amongst probiotic products. Although 16s and *rpoA* gene sequences have been extensively used to classify *Lactobacillus* strains, identification of lactobacilli at species and/or subspecies level using these gene sequences is proven to be difficult. Therefore, analysis of other gene sequences might be helpful as alternative genomic markers to the aforementioned gene sequencing techniques, and may have a higher discriminatory power for reliable identification of *Lactobacillus* spp.

## Figures and Tables

**Table 1:** Details of tested probiotic products.

Sample Code	Product Description	Days to Expire	Claimed Culture (s)	Country of Origin
1	Stirred yoghurt	10	*Bifidobacterium, Lb. acidophilus,* *Streptococcus thermophilus*	UK
2	Organic natural yoghurt	12	*Lb. acidophilus, Bifidobacterium*	UK
3	Natural fresh and mild yoghurt	17	*Lb. acidophilus, B. longum,* *S. thermophilus*	UK
4	Fruit yoghurt	13	*Lb. acidophilus, Bifidobacteria*	UK
5	Thick and creamy yoghurt	11	*Bifidobacterium, Lb. acidophilus,* *S. thermophilus*	UK
6	Fruit yoghurt	13	*B. animalis* subsp.*lactis* *Lb. acidophilus*	UK
7	Natural goat yoghurt	25	*B. longum, Lb. acidophilus*	UK
8	Goat fruit yoghurt	26	*S. thermophilus, Lb. casei*	UK
9	Natural Greek style	15	*Lb. acidophilus, Lb. bulgaricus,* *S. thermophilus*	UK
10	Fruit yoghurt	11	*Lb. bulgaricus, Lb. acidophilus,* *S. thermophilus,* *Bifidobacterium*	UK
11	Fat-free yoghurt drink	19	*Lb. casei*	Denmark
12	Bio pouring yoghurt	11	Probiotic	UK
13	Fruit yoghurt drink	4	*Lb. casei*	UK
14	Fermented milk drink	23	*Lb. casei* Shirota	UK
15	Fruit yoghurt	19	probiotic	Ireland
16	Gout milk yoghurt	17	*Lb. acidophilus, Lb. bulgaricus,* *S. thermophilus, Bifidobacterium*	UK
17	Fruit yoghurt	14	*Lb. acidophilus,* *Bifidobacterium,* *S. thermophilus*	UK
18	Fruit yoghurt smoothie	20	*Yoghurt culture, Lb. acidophilus,* *Bifidobacterium*	UK
19	Yoghurt drink	11	*Lb. casei*	UK
20	Fruit yoghurt	11	*S. thermophilus, Lb. acidophilus,* *Bifidobacterium,* *Lb. casei*	UK
21	Live natural yoghurt	23	*Lb. acidophilus,* *Lb. casei,* *Bifidobacterium*	Germany
22	Fromage frais blanc	27	*Bifidobacterium,* *Lb. acidophilus*	France
23	Yoghurt	12	Sainsbury’s probiotic bacteria	UK
24	Yoghurt drink	26	*Lb. acidophilus* La5	UK
25	Yoghurt drink	19	Sainsbury’s probiotic bacteria, *Lb. casei*	UK
26	Probiotic yoghurt selection	11	probiotic	UK
27	Fermented soya drink	11	*Bifidus,* *Lb. acidophilus*	France
28	Organic kefir	17	Probiotic	Belgium
29	Natural probiotic drink	19	Rich in probiotic	UK
30	Fruit layer yoghurt	20	*Lb. acidophilus* La5 *B. animalis* subsp. *lactis* BB12	Germany
31	Probiotic yoghurt	15	probiotic	Germany
32	Stirred yoghurt	10	*B. animalis* subsp. *lactis* BB12 *L. acidophilus*	Germany
33	Stirred yoghurt	8	*Lb.* lc1	Germany
34	Fruit yoghurt	4	*Lb. casei*	Germany
35	Probiotic yoghurt drink	3	*B. animalis* subsp. *lactis* BB12 *Lb. acidophilus* La5 *Lb. casei*	Germany
36	Fruit yoghurt	7	*Lb. casei*	Germany

**Table 2:** The identity of probiotic lactobacilli isolated from commercial fermented milks by sequence analysis of 16s rRNA and *rpoA* genes, compared with claimed cultures by manufacturers, as well as the initial, final, and changes in the pH of the tested products.

Sample Code	Claimed Culture (s)	Identified Isolate (s)	Initial pH *	Final pH **	Δ pH
1	*Bifidobacterium,* *Lb. acidophilus,* *S. thermophilus*	*Lb. acidophilus*	4.16	3.99	0.17
2	*Lb. acidophilus,* *Bifidobacterium*	*Lb. acidophilus*	4.10	4.00	0.10
3	*Lb. acidophilus,* *B. longum,* *S. thermophilus*	*Lb. acidophilus*	4.12	4.01	0.11
4	*Lb. acidophilus,* *Bifidobacteria*	*Lb. acidophilus*	4.05	3.92	0.13
5	*Bifidobacterium,* *Lb. acidophilus,* *S. thermophilus*	*Lb. acidophilus*	4.08	4.01	0.07
6	*B. animalis* subsp. *lactis* *Lb. acidophilus*	*Lb. acidophilus*	4.22	4.10	0.12
7	*B. longum,* *Lb. acidophilus*	*Lb. johnsonii*	3.95	3.66	0.29
8	*S. thermophilus,* *Lb. casei*	*Lb. casei/paracasei*	3.80	3.80	0
9	*Lb. acidophilus,* *Lb. bulgaricus,* *S. thermophilus*	*Lb. acidophilus*	4.28	4.20	0.08
10	*Lb. bulgaricus,* *Lb. acidophilus,* *S. thermophilus,* *Bifidobacterium*	*Lb. acidophilus*	4.70	4.61	0.09
11	*Lb. casei*	*Lb. casei/paracasei*	4.06	4.01	0.05
12	Probiotic	*Lb. acidophilus*	3.96	3.94	0.02
13	*Lb. casei*	*Lb. acidophilus* *Lb. casei/paracasei*	4.24	4.02	0.22
14	*Lb. casei* Shirota	*Lb. casei/paracasei*	3.76	3.62	0.14
15	probiotic	*Lb. acidophilus*	3.95	3.95	0
16	*Lb. acidophilus,* *Lb. bulgaricus,* *S. thermophilus,* *Bifidobacterium*	*Lb. acidophilus*	4.16	3.99	0.17
17	*Lb. acidophilus,* *Bifidobacterium,* *S. thermophilus*	*Lb. acidophilus*	3.95	3.86	0.09
18	*Yoghurt culture,* *Lb. acidophilus,* *Bifidobacterium*	*Lb. acidophilus*	3.85	3.61	0.24
19	*Lb. casei*	*Lb. casei/paracasei* *Lb. acidophilus*	4.06	3.97	0.09
20	*S. thermophilus,* *Lb. acidophilus,* *Bifidobacterium,* *Lb. casei*	*Lb. casei/paracasei* *Lb. acidophilus*	3.97	3.55	0.42
21	*Lb. acidophilus,* *Lb. casei,* *Bifidobacterium*	*Lb. johnsonii*	4.21	3.94	0.27
22	*Bifidobacterium,* *Lb. acidophilus*	*Lb. acidophilus*	4.24	4.21	0.03
23	Sainsbury’s probiotic bacteria	*Lb. acidophilus* *S.* *thermophilus*	3.8	3.79	0.01
24	*Lb. acidophilus* La5	*Lb. casei/paracasei* *Lb. acidophilus*	4.05	3.98	0.07
25	Sainsbury’s probiotic bacteria, *Lb. casei*	*Lb. acidophilus* *Lb.* *casei/paracasei*	3.76	3.46	0.3
26	probiotic	*Lb. acidophilus*	3.92	3.81	0.11
27	*Bifidus,* *Lb. acidophilus*	No growth	4.07	3.78	0.29
28	Probiotic	*Lb. helveticus/gallinarum/suntoryeus*	3.99	3.85	0.14
29	Rich in probiotic	*Lb. acidophilus*	4.45	4.29	0.16
30	*Lb. acidophilus* La5 *B. animalis* subsp. *lactis* BB12	*Lb. acidophilus* *Lb. casei/paracasei*	3.9	3.82	0.08
31	probiotic	*Lb. acidophilus*	3.99	3.77	0.22
32	*B. animalis* subsp. *lactis* BB12 *L. acidophilus*	*Lb. acidophilus*	3.97	3.95	0.02
33	*Lb.* lc1	*Lb. johnsonii*	4.16	4.11	0.05
34	*Lb. casei*	*Lb. helveticus/gallinarum/suntoryeus*	3.92	3.85	0.07
35	*B. animalis* subsp. *lactis* BB12 *Lb. acidophilus* La5 *Lb. casei*	*Lb. acidophilus* *Lb.* *casei/paracasei*	4.04	3.94	0.1
36	*Lb. casei*	*Lb. casei/paracasei* *Lb. helveticus/gallinarum/suntoryeus*	4.05	3.88	0.17

* Initial pH was measured upon samples’ arrival to the lab. ** Final pH was measured on the expiry date.

## Data Availability

Not applicable.
